# Association between total cholesterol and all-cause mortality in oldest old: a national longitudinal study

**DOI:** 10.3389/fendo.2024.1405283

**Published:** 2024-06-13

**Authors:** Fan Hu, Zhiqiang Wang, Yujie Liu, Ying Gao, Shangbin Liu, Chen Xu, Ying Wang, Yong Cai

**Affiliations:** Public Health Department, International Institute of Medicine, Tongren Hospital, Shanghai Jiao Tong University School of Medicine, Shanghai, China

**Keywords:** total cholesterol, all-cause mortality, oldest old, Cox regression, cohort study

## Abstract

**Background:**

A common sense is that lower serum cholesterol levels are better. However, a growing number of researches have questioned this especially for the oldest old. The current study was to assess the association between total cholesterol and all-cause mortality in a group of people aged 85 years old and over.

**Methods:**

We selected 903 Chinese old participants who aged ≥85 years from the Chinese Longitudinal Healthy Longevity Survey(CLHLS) at baseline in 2012. The participants were followed up until death or until December 31, 2014. The outcome was all-cause mortality. The univariate and multivariate Cox regression analyses were used to estimate risk levels of all-cause mortality. We stratified the participants into three groups (<3.40, 3.40–4.39, ≥4.39 mmol/L) based on the restricted cubic splines methods. The survival probability according to total cholesterol category was calculated using the Kaplan-Meier curves, and the log-rank test was performed to analyze differences between the groups.

**Results:**

During the follow-up of three years, 282 participants died, 497 survived and 124 lost to follow-up. There was significant relationship between the total cholesterol and lower risk of all-cause mortality in the multivariable Cox regression analysis (HR=0.88, 95% CI: 0.78–1.00). Based on the restricted cubic splines methods, the total cholesterol was converted from a continuous variable to a categorical variable. The populations were divided into three groups (<3.40, 3.40–4.39, ≥4.39 mmol/L) according to the total cholesterol categorized by cutoff values. Compared to the total cholesterol level of <3.40 mmol/L, populations in the total cholesterol level of 3.40–4.39 mmol/L (HR = 0.72, 95% CI: 0.53–0.97) and ≥4.39 mmol/L (HR = 0.71, 95% CI: 0.52–0.96) groups had lower all-cause mortality in multivariate Cox regression analysis and higher survival probability in survival analysis. When two groups were divided, similar results were found among the populations in the total cholesterol level of ≥3.40 mmol/L compared to the populations in the total cholesterol level of <3.40 mmol/L groups.

**Conclusion:**

In oldest old aged 85 and older, serum total cholesterol levels are inversely associated with all-cause mortality. This study suggested that total cholesterol should be maintained to acceptable levels (≥ 3.40 mmol/L) in oldest old to achieve longevity.

## Introduction

The global population is living longer and living more years in good health. Global life expectancy at birth has increased from 66.8 years in 2000 to 72.0 years in 2020 (1), and life expectancy per capita in China reached 77.9 years in 2020 (2). Population aging is intensifying: the proportion of the global population aged over 60 years is predicted to nearly double from 12% to 22% between 2015 and 2050 (3). Population aging has led to a rapid growth in the global numbers of oldest-old individuals, defined as those aged over 80 years. Therefore, it is important to ensure that this population experiences healthy longevity.

Cardiovascular diseases are the leading cause of death worldwide (4). Studies of middle-aged and early-old people have found that high cholesterol levels are positively associated with death from ischemic heart disease, and that lowering lipid levels improves health (5, 6). The National Cholesterol Education Program guideline recommends total cholesterol (TC) levels <200 mg/dL as acceptable for healthy persons, levels of 200–239 mg/dL as borderline high, and levels of ≥240 mg/dL as high. A high plasma concentration of cholesterol is one of the main risk factors for atherosclerosis, which is a major cause of cardiovascular disease (7). However, cholesterol plays an important part in regulating many cellular processes, from membrane fluidity and permeability to gene transcription, and is the basis of all steroid hormones and vitamin D analogs (8). Physiological functions change with age and the association between cholesterol level and all-cause mortality may differ according to age. For example, a classic study showed that high TC concentrations are protectively associated with longevity in people older than 85 years (9). The Honolulu Heart Program showed that long-term low cholesterol concentrations actually increase the risk of death (10). A 10-year longitudinal study in Korea found a U-shaped association between TC and all-cause mortality in individuals aged 75–99 years, and showed that the TC level associated with the lowest mortality risk was 210–249 mg/dL, which exceeds the recommended level (11). However, data on the effect of TC among oldest-old adults is scarce and sometimes conflicting. Few studies have established a lower reference limit for cholesterol level, despite the negative association between cholesterol level and all-cause mortality. Furthermore, previous research has lacked large samples and has not examined the older population in China.

The present study used data from the nationwide Chinese Longitudinal Healthy Longevity Survey (CLHLS), which collected health information from more than 10,000 people aged over 65 years. Questionnaire data (2011) and plasma biochemical parameters (2012) from the fifth follow-up survey were analyzed. The aim of this study was to extend knowledge about the association between TC level and all-cause mortality among people aged ≥85 years (which we defined as oldest old) to explore how TC levels affect longevity and to determine a lower limit of safe TC for this population.

## Methods

### Study population

The study population was drawn from the 2011/2012 and 2014 CLHLS. The CLHLS is the first and largest nationwide, community-based, longitudinal prospective survey in China (12). The CLHLS was designed to investigate the determinants of health and longevity among older adults (aged ≥65 years), particularly oldest-old adults (aged ≥85 years). The CLHLS randomly selected participants from counties or cities in 22 provinces across China, covering about 85% of the total population in China (13). The CLHLS began in 1998, and follow-up surveys were conducted in 2000, 2002, 2005, 2008/2009, 2011/2012, 2014, and 2017/2018 (14). The CLHLS collects many different types of information about older people, including demographics, lifestyle, diet, health status, and daily activities. In 2012, a biomarker substudy was launched in eight longevity regions: Sanshui in Guangdong Province, Yongfu in Guangxi Autonomous Area, Chengmai in Hainan Province, Xiayi in Henan Province, Zhongxiang in Hubei Province, Mayang in Hunan Province, Laizhou in Shandong Province, and Rudong in Jiangsu Province. The CLHLS study was approved by the research ethics committee of Peking University (IRB00001052–13074). All participants provided written informed consent.

**Figure 1:**
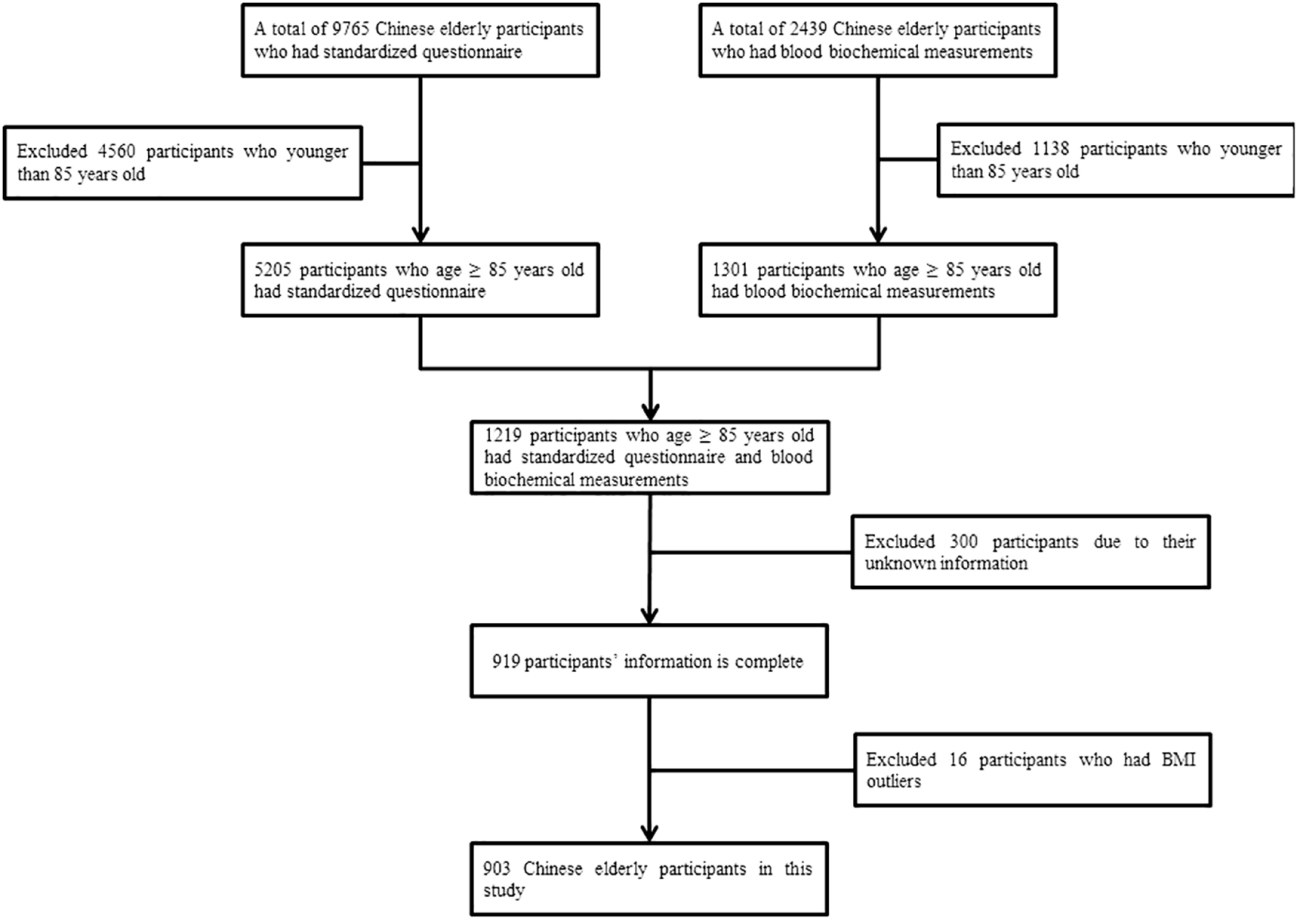
Flow chart on the selection of study population.

Initially, a total of 9765 old Chinese participants were included in the study. Using the following exclusion criteria, we retained 903 participants. We excluded (1) participants younger than 85 years; (2) participants with missing information; (3) participants who had body mass index data outliers. **Figure 1** shows the study population flow chart.

### Data collection

We collected information using a standardized questionnaire administered during a household interview. The information collected included data on demographics, health-related lifestyle factors, and self-reported medical history. Demographic data comprised age, sex, and residence. Health-related lifestyle data comprised diet (frequency of consuming fresh fruit, vegetables, meat, fish, eggs, beans, sugar, tea, milk products, nut products, and vitamins), smoking, drinking alcohol, and exercise. Self-reported medical history data comprised presence of hypertension, diabetes, heart disease, stroke or cerebrovascular disease, cancer, blood disease, and chronic nephritis. After the household interview, all participants were asked to undergo anthropometric measurements, which included systolic blood pressure (mmHg), diastolic blood pressure (mmHg), and body mass index (kg/m^2^). The blood biochemistry tests analyzed in this study included blood urea nitrogen (mmol/L), plasma creatine (mmol/L), uric acid (µmol/L), plasma glucose (mmol/L), TC (mmol/L), high-density lipoprotein cholesterol (mmol/L), low-density lipoprotein cholesterol (mmol/L), triglycerides (mmol/L), and vitamin B12 (pmol/L). All the information, including TC levels, which is the main exposure variable, was collected in the 2011/2012 wave.

### Biochemical measurements

Fasting venous blood samples were collected by trained medical personnel from all willing participants who had fasted overnight; 5 mL of fasting venous blood was collected in heparin anticoagulant vacuum tubes and centrifuged at 20°C and 2500 rpm for 10 minutes. The plasma was isolated and frozen at −20°C, shipped cold chained to the central laboratory at Capital Medical University in Beijing, and stored at −80°C until biochemical analysis. Blood urea nitrogen, plasma creatine, uric acid, plasma glucose, TC, high-density lipoprotein cholesterol, low-density lipoprotein cholesterol, triglycerides, and vitamin B12 were measured using an automatic biochemistry analyzer (7180; Hitachi, Roche, Basel, Switzerland).

### Study outcome during prospective follow-up

The study outcome was all-cause mortality from January 1, 2012, to December 31, 2014. Mortality status was obtained from a CLHLS publicly available dataset, which recorded the vital status of survey participants from baseline to December 31, 2014. If a participant missed the follow-up visit, survival time was defined as the interval between baseline and December 31, 2014.

### Statistical analysis

Continuous variables are presented as means ± standard deviations; categorical variables are presented as frequencies (%). Univariate and multivariate Cox regression analysis were used to estimate hazard ratios (HRs) and 95% confidence interval (CI) values for all-cause mortality. Variables with *P* values <0.05 in the univariate analysis were considered potential influencing factors and included in the multivariate Cox regression analysis. This regression analysis included TC, age, diet (consumption of fresh fruit, fish, eggs, sugar, and nut products), presence of hypertension, systolic blood pressure, and blood urea nitrogen. In this study, we also explored the potentially non-linear relationship between TC and all-cause mortality. To balance best fit and overfitting in the main splines for all-cause mortality, a knot number between three and seven was chosen as the maximum value for the squared multiple correlation coefficient R^2^ (15). We used multivariate Cox regression models with the restricted cubic splines method, with seven TC knots. To analyze further the relationship between TC and all-cause mortality, we stratified participants into three groups (<3.40, 3.40–4.39, ≥4.39 mmol/L) based on the restricted cubic splines method. To explore differences in the effect of TC on the all-cause mortality for different characteristics, participants were classified into subgroups by age, consumption of fresh fruit, fish, eggs, sugar. The survival probability according to TC category was calculated using the Kaplan–Meier curve, and the log-rank test was used to analyze differences between the groups. The statistical analysis was performed using the R 4.03 statistical package (The R Foundation for Statistical Computing). A two-sided *P* value of <0.05 was considered to indicate statistical significance.

## Results

### Baseline characteristics

The baseline characteristics of the study population are summarized in **Table 1**. Data for 903 participants were analyzed. The mean age of the study population was 95.77 ± 6.40 years. During the 3-year follow-up, 282 participants died, 497 survived, and 124 were lost to follow-up.

**Table 1:** Baseline characteristics of the study population.

Characteristics	Total (n=903)	Survivors (n=497)	Death (n=282)	Lost to follow-up (n=124)
Age (years)	95.77 ± 6.40	94.96 ± 6.53	97.27 ± 6.02	95.65 ± 6.19
Sex				
Male	287 (31.78)	149 (29.98)	95 (33.69)	43 (34.68)
Female	616 (68.22)	348 (70.02)	187 (66.31)	81 (65.32)
Category of residence				
City	34 (3.77)	16 (3.22)	10 (3.55)	8 (6.45)
Town	869 (96.23)	481 (96.78)	272 (96.45)	116 (93.55)
Eat fresh fruit				
No	619 (68.55)	315 (63.38)	208 (73.76)	96 (77.42)
Yes	284 (31.45)	182 (36.62)	74 (26.24)	28 (22.58)
Eat vegetables				
No	182 (20.16)	83 (16.70)	67 (23.76)	32 (25.81)
Yes	721 (79.84)	414 (83.30)	215 (76.24)	92 (74.19)
Exercise				
No	799 (88.48)	447 (89.94)	257 (91.13)	95 (76.61)
Yes	104 (11.52)	50 (10.06)	25 (8.87)	29 (23.39)
Eat meat				
No	50 (5.54)	26 (5.23)	18 (6.38)	6 (4.84)
Sometimes	555 (61.46)	317 (63.78)	169 (59.93)	69 (55.65)
Yes	298 (33.00)	154 (30.99)	95 (33.69)	49 (39.52)
Eat fish				
No	135 (14.95)	56 (11.27)	63 (22.34)	16 (12.90)
Sometimes	697 (77.19)	404 (81.29)	202 (71.63)	91 (73.39)
Yes	71 (7.86)	37 (7.44)	17 (6.03)	17 (13.71)
Eat eggs				
No	73 (8.08)	40 (8.05)	16 (5.67)	17 (13.71)
Sometimes	544 (60.24)	289 (58.15)	167 (59.22)	88 (70.97)
Yes	286 (31.67)	168 (33.80)	99 (35.11)	19 (15.32)
Eat beans				
No	86 (9.52)	47 (9.46)	22 (7.80)	17 (13.71)
Sometimes	739 (81.84)	409 (82.29)	233 (82.62)	97 (78.23)
Yes	78 (8.64)	41 (8.25)	27 (9.57)	10 (8.06)
Eat sugar				
No	236 (26.14)	129 (25.96)	67 (23.76)	40 (32.26)
Sometimes	530 (58.69)	301 (60.56)	155 (54.96)	74 (59.68)
Yes	137 (15.17)	67 (13.48)	60 (21.28)	10 (8.06)
Drink tea				
No	604 (66.89)	326 (65.59)	194 (68.79)	84 (67.74)
Sometimes	168 (18.60)	90 (18.11)	58 (20.57)	20 (16.13)
Yes	131 (14.51)	81 (16.30)	30 (10.64)	20 (16.13)
Eat milk products				
No	399 (44.19)	216 (43.46)	118 (41.84)	65 (52.42)
Sometimes	404 (44.74)	225 (45.27)	131 (46.45)	48 (38.71)
Yes	100 (11.07)	56 (11.27)	33 (11.70)	11 (8.87)
Eat nut products				
No	595 (65.89)	319 (64.19)	198 (70.21)	78 (62.90)
Sometimes	299 (33.11)	174 (35.01)	79 (28.01)	46 (37.10)
Yes	9 (1.00)	4 (0.80)	5 (1.77)	0
Eat vitamins				
No	789 (87.38)	426 (85.71)	248 (87.94)	115 (92.74)
Sometimes	88 (9.75)	54 (10.87)	26 (9.22)	8 (6.45)
Yes	26 (2.88)	17 (3.42)	8 (2.84)	1 (0.81)
Smoke				
No	754 (83.50)	411 (82.70)	241 (85.46)	102 (82.26)
Yes	149 (16.50)	86 (17.30)	41 (14.54)	22 (17.74)
Drink				
No	775 (85.83)	424 (85.31)	242 (85.82)	109 (87.90)
Yes	128 (14.17)	73 (14.69)	40 (14.18)	15 (12.10)
Hypertension				
No	682 (75.53)	371 (74.65)	229 (81.21)	82 (66.13)
Yes	221 (24.47)	126 (25.35)	53 (18.79)	42 (33.87)
Diabetes				
No	897 (99.34)	491 (98.79)	282 (100.00)	124 (100.00)
Yes	6 (0.66)	6 (1.21)	0	0
Heart disease				
No	850 (94.13)	463 (93.16)	267 (94.68)	120 (96.77)
Yes	53 (5.87)	34 (6.84)	15 (5.32)	4 (3.23)
Stroke or cerebrovascular disease				
No	836 (92.58)	458 (92.15)	262 (92.91)	116 (93.55)
Yes	67 (7.42)	39 (7.85)	20 (7.09)	8 (6.45)
Cancer				
No	900 (99.67)	496 (99.80)	280 (99.29)	124 (100.00)
Yes	3 (0.33)	1 (0.20)	2 (0.71)	0
Blood disease				
No	897 (99.34)	494 (99.40)	279 (98.94)	124 (100.00)
Yes	6 (0.66)	3 (0.60)	3 (1.06)	0
Chronic nephritis				
No	901 (99.78)	496 (99.80)	281 (99.65)	124 (100.00)
Yes	2 (0.22)	1 (0.20)	1 (0.35)	0
SBP (mmHg)	142.78 ± 24.45	144.35 ± 23.91	139.47 ± 24.50	144.02 ± 25.88
DBP (mmHg)	80.18 ± 12.51	80.49 ± 12.24	80.21 ± 12.87	78.88 ± 12.81
BMI (kg/m^2^)	20.10 ± 3.97	20.37 ± 3.91	19.76 ± 4.07	19.74 ± 3.92
Blood Urea Nitrogen (mmol/L)	7.24 ± 2.44	7.11 ± 2.19	7.50 ± 2.77	7.16 ± 2.56
Plasma creatine (mmol/L)	84.51 ± 34.79	83.55 ± 32.67	83.41 ± 38.21	90.86 ± 34.50
Urea acid (umol/L)	288.54 ± 85.18	287.72 ± 82.48	285.62 ± 88.89	298.50 ± 87.18
Plasma glucose (mmol/L)	4.65 ± 1.68	4.57 ± 1.44	4.79 ± 1.88	4.63 ± 2.03
Total cholesterol (mmol/L)	4.19 ± 1.00	4.28 ± 0.98	4.06 ± 0.98	4.10 ± 1.09
HDL cholesterol (mmol/L)	1.29 ± 0.34	1.32 ± 0.35	1.26 ± 0.31	1.23 ± 0.35
LDL cholesterol (mmol/L)	2.50 ± 0.83	2.55 ± 0.81	2.42 ± 0.82	2.48 ± 0.88
Triglyceride (mmol/L)	0.89 ± 0.49	0.92 ± 0.54	0.84 ± 0.43	0.85 ± 0.41
Vitamin B12 (pmol/L)	374.00 ± 202.14	383.75 ± 204.58	355.70 ± 200.24	376.58 ± 195.20

Data are presented as mean ± standard deviation for continuous variables and n (%) for categorical variables.

SBP, systolic blood pressure; DBP, diastolic blood pressure; BMI, body mass index; HDL, high-density lipoprotein; LDL, low-density lipoprotein.

### Association between total cholesterol and all-cause mortality

When TC was used as a continuous variable, there was a significant association between TC and lower risk of all-cause mortality (HR = 0.85, 95% CI: 0.76–0.96; *P* = 0.011) in the univariate Cox regression analysis. All variables that were significant in the univariate Cox regression analysis were incorporated into the multivariate Cox regression model to assessed the independent predictors of all-cause mortality (**Table 2**), including age, fresh fruit consumption, fish consumption, egg consumption, sugar consumption, nut product consumption, hypertension, systolic blood pressure, and blood urea nitrogen. After adjusting for these factors, TC remained the primary independent risk factor for all-cause mortality (HR = 0.88, 95% CI: 0.78–1.00; *P* = 0.043). These results suggest that TC has a protective effect.

**Table 2:** Hazard ratios for all-cause mortality in the univariate and multivariate Cox regression analysis.

Characteristics	Univariate			Multivariate		
	HR	95%CI	*p* value	HR	95%CI	*p* value
Age(years)						
<100	Reference			Reference		
≥100	1.49	1.18–1.88	0.001	1.40	1.10–1.77	0.006
Sex						
Male	Reference			Not included		
Female	0.91	0.71–1.16	0.445			
Category of residence						
City	Reference			Not included		
Town	1.07	0.57–2.02	0.823			
Eat fresh fruit						
No	Reference			Reference		
Yes	0.75	0.57–0.97	0.030	0.75	0.57–0.98	0.038
Eat vegetables						
No	Reference			Not included		
Yes	0.80	0.61–1.06	0.120			
Exercise						
No	Reference			Not included		
Yes	0.70	0.46–1.05	0.088			
Eat meat						
No	Reference			Not included		
Sometimes	0.81	0.50–1.31	0.383			
Yes	0.87	0.52–1.44	0.582			
Eat fish						
No	Reference			Reference		
Sometimes	0.54	0.41–0.71	<0.001	0.54	0.40–0.73	<0.001
Yes	0.43	0.25–0.73	0.002	0.43	0.25–0.74	0.002
Eat eggs						
No	Reference			Reference		
Sometimes	1.46	0.87–2.44	0.147	1.66	0.98–2.81	0.059
Yes	1.69	1.00–2.87	0.050	1.84	1.07–3.16	0.029
Eat beans						
No	Reference			Not included		
Sometimes	1.28	0.82–1.98	0.274			
Yes	1.45	0.83–2.55	0.194			
Eat sugar						
No	Reference			Reference		
Sometimes	1.01	0.76–1.35	0.945	1.02	0.76–1.37	0.894
Yes	1.75	1.24–2.48	0.002	1.66	1.14–2.39	0.007
Drink tea						
No	Reference			Not included		
Sometimes	1.08	0.81–1.45	0.593			
Yes	0.69	0.47–1.01	0.054			
Eat milk products						
No	Reference			Not included		
Sometimes	1.12	0.87–1.44	0.365			
Yes	1.17	0.79–1.72	0.435			
Eat nut products						
No	Reference			Reference		
Sometimes	0.75	0.58–0.98	0.032	0.86	0.66–1.13	0.279
Yes	1.73	0.71–4.20	0.227	2.01	0.80–5.07	0.138
Eat vitamins						
No	Reference			Not included		
Sometimes	0.91	0.61–1.37	0.662			
Yes	0.93	0.46–1.87	0.831			
Smoke						
No	Reference			Not included		
Yes	0.84	0.61–1.18	0.317			
Drink						
No	Reference			Not included		
Yes	1.03	0.73–1.43	0.877			
Hypertension						
No	Reference			Reference		
Yes	0.66	0.49–0.89	0.006	0.73	0.53–1.02	0.063
Diabetes						
No	Reference			Not included		
Yes	0.00	0.00–1.03E225	0.965			
Heart disease						
No	Reference			Not included		
Yes	0.87	0.52–1.47	0.603			
Stroke or cerebrovascular disease						
No	Reference			Not included		
Yes	0.91	0.58–1.44	0.694			
Cancer						
No	Reference			Not included		
Yes	2.79	0.69–11.20	0.148			
Blood disease						
No	Reference			Not included		
Yes	1.86	0.60–5.81	0.284			
Chronic nephritis						
No	Reference			Not included		
Yes	1.39	0.20–9.92	0.739			
SBP(mmHg)	0.99	0.99–1.00	0.010	1.00	0.99–1.00	0.441
DBP(mmHg)	1.00	0.99–1.01	0.946	Not included		
BMI(kg/m^2^)	0.97	0.94–1.00	0.076	Not included		
Blood Urea Nitrogen(mmol/L)	1.05	1.01–1.10	0.018	1.04	0.99–1.09	0.113
Plasma creatine(mmol/L)	1.00	1.00–1.00	0.524	Not included		
Urea acid(umol/L)	1.00	1.00–1.00	0.583	Not included		
Plasma glucose(mmol/L)	1.06	0.99–1.13	0.090	Not included		
Total cholesterol(mmol/L)	0.85	0.76–0.96	0.011	0.88	0.78–1.00	0.043
HDL cholesterol(mmol/L)	0.76	0.54–1.07	0.114	Not included		
LDL cholesterol(mmol/L)	0.87	0.75–1.01	0.062	Not included		
Triglyceride(mmol/L)	0.77	0.59–1.01	0.058	Not included		
Vitamin B12(pmol/L)	1.00	1.00–1.00	0.081	Not included		

SBP, systolic blood pressure; DBP, diastolic blood pressure; BMI, body mass index; HDL, high-density lipoprotein; LDL, low-density lipoprotein; HR, hazard ratio; CI, confidence interval.

### A non-linear relationship between total cholesterol and all-cause mortality

**Figure 2:**
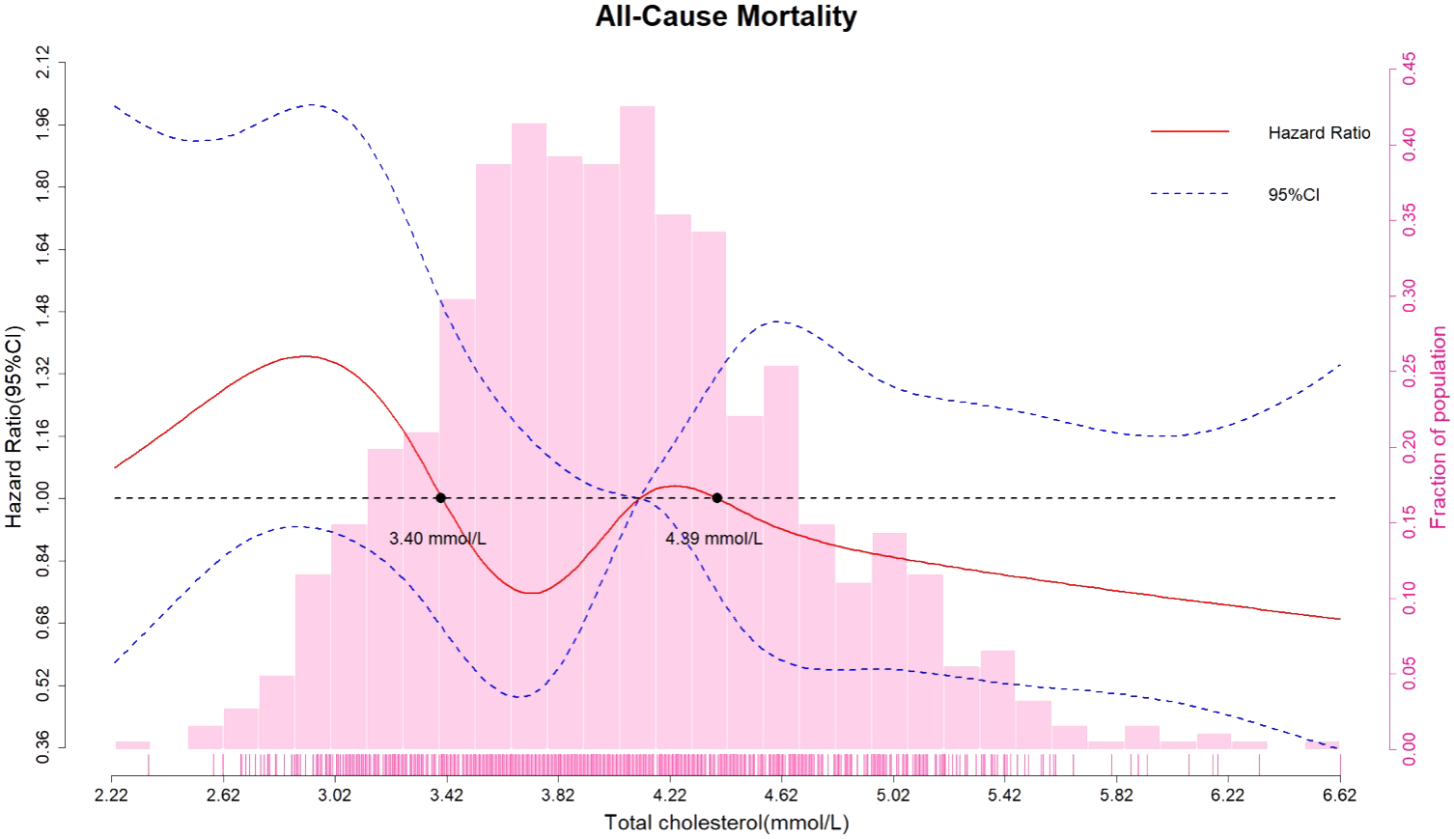
Association between all-cause mortality and total cholesterol on a continuous scale. Hazard ratios (solid red line) and 95% confidence intervals (dashed blue lines) from multivariate Cox regression models using restricted cubic splines method. Reference lines for no association are indicated by the dashed black line (hazard ratio of 1.00). Multivariable adjustment was performed for age, fresh fruit consumption, fish consumption, egg consumption, sugar consumption, nut product consumption, hypertension, systolic blood pressure, and blood urea nitrogen. CI, confidence interval.

To further evaluate a possible non-linear relationship, the restricted cubic splines method was used to investigate the association between all-cause mortality and TC on a continuous scale. As shown in **Figure 2**, the multivariate Cox regression models with restricted cubic splines method indicated that the relationship between TC and all-cause mortality might be non-linear. After adjusting for various potential confounders, TC was inversely associated with the risk of all-cause mortality. The optimal TC cutoff values for all-cause mortality were 3.40 mmol/L and 4.39 mmol/L. Lower TC levels (<3.40 mmol/L) were significantly associated with an increased risk of all-cause mortality. However, higher TC levels (≥4.39 mmol/L) were significantly associated with a reduced risk of all-cause mortality. However, the limitation is due to the relatively small sample size and limitations of the modelling method, the confidence intervals of the spline curves included 1 at all TC levels, suggesting that the association between TC and mortality may not be significant, and therefore our derivation of the optimal cut-off point may have been biased.

### All-cause mortality according to total cholesterol categorized by cutoff values

Multivariate Cox regression analysis was used to examine the relationship between TC category and all-cause mortality. Based on the restricted cubic splines method, TC was converted from a continuous variable to a categorical variable. The populations were divided into three groups according to TC categorized by cutoff values. Compared with participants with TC levels of <3.40 mmol/L, participants with TC levels of 3.40–4.39 mmol/L (HR = 0.72, 95% CI: 0.53–0.97; *P* = 0.029) and ≥4.39 mmol/L (HR = 0.71, 95% CI: 0.52–0.96; *P* = 0.029) had lower all-cause mortality according to the multivariate Cox regression analysis adjusted for age, fresh fruit consumption, fish consumption, egg consumption, sugar consumption, nut product consumption, hypertension, systolic blood pressure, and blood urea nitrogen (**Table 3**). In addition, participants with TC levels of ≥3.40 mmol/L (HR = 0.71, 95% CI: 0.54–0.93; *P* = 0.014) had a significantly lower risk of all-cause mortality than participants with TC levels of <3.40 mmol/L (**Table 4**).

**Table 3:** Hazard ratios for all-cause mortality according to total cholesterol category: multivariate Cox regression analysis.

Characteristics	Multivariate		
	HR	95%CI	*p* value
Total cholesterol(mmol/L)			
<3.40	Reference		
3.40–4.39	0.72	0.53–0.97	0.029
≥4.39*	0.71	0.52–0.96	0.029
Age(years)			
<100	Reference		
≥100	1.40	1.11–1.78	0.005
Eat fresh fruit			
No	Reference		
Yes	0.74	0.56–0.97	0.029
Eat fish			
No	Reference		
Sometimes	0.54	0.40–0.72	<0.001
Yes	0.41	0.24–0.72	0.002
Eat eggs			
No	Reference		
Sometimes	1.67	0.99–2.83	0.057
Yes	1.83	1.06–3.16	0.029
Eat sugar			
No	Reference		
Sometimes	1.03	0.77–1.39	0.835
Yes	1.69	1.17–2.45	0.005
Eat nut products			
No	Reference		
Sometimes	0.85	0.65–1.12	0.250
Yes	1.84	0.73–4.60	0.195
Hypertension			
No	Reference		
Yes	0.75	0.54–1.03	0.076
SBP (mmHg)	1.00	0.99–1.00	0.446
Blood Urea Nitrogen (mmol/L)	1.04	0.99–1.09	0.109

SBP, systolic blood pressure; HR, hazard ratio; CI, confidence interval.

*There was no significant difference in all-cause mortality between the two groups (3.40–4.39 mmol/L and ≥4.39 mmol/L) after adjusting for age, fresh fruit consumption, fish consumption, egg consumption, sugar consumption, nut product consumption, hypertension, SBP, and blood urea nitrogen.

**Table 4:** Hazard ratios for all-cause mortality according to total cholesterol ≥3.40 mmol/L compared with <3.40 mmol/L: multivariate Cox regression analysis.

Characteristics	Multivariate		
	HR	95%CI	*p* value
Total cholesterol(mmol/L)			
<3.40	Reference		
≥3.40	0.71	0.54–0.93	0.014
Age(years)			
<100	Reference		
≥100	1.40	1.11–1.78	0.005
Eat fresh fruit			
No	Reference		
Yes	0.74	0.56–0.97	0.028
Eat fish			
No	Reference		
Sometimes	0.54	0.40–0.72	<0.001
Yes	0.41	0.24–0.72	0.002
Eat eggs			
No	Reference		
Sometimes	1.67	0.99–2.83	0.056
Yes	1.83	1.06–3.16	0.029
Eat sugar			
No	Reference		
Sometimes	1.03	0.77–1.39	0.837
Yes	1.70	1.17–2.46	0.005
Eat nut products			
No	Reference		
Sometimes	0.85	0.65–1.12	0.247
Yes	1.83	0.73–4.58	0.196
Hypertension			
No	Reference		
Yes	0.75	0.54–1.03	0.076
SBP (mmHg)	1.00	0.99–1.00	0.441
Blood Urea Nitrogen(mmol/L)	1.04	0.99–1.09	0.109

SBP, systolic blood pressure; HR, hazard ratio; CI, confidence interval.

### Subgroup analysis

**Figure 3:**
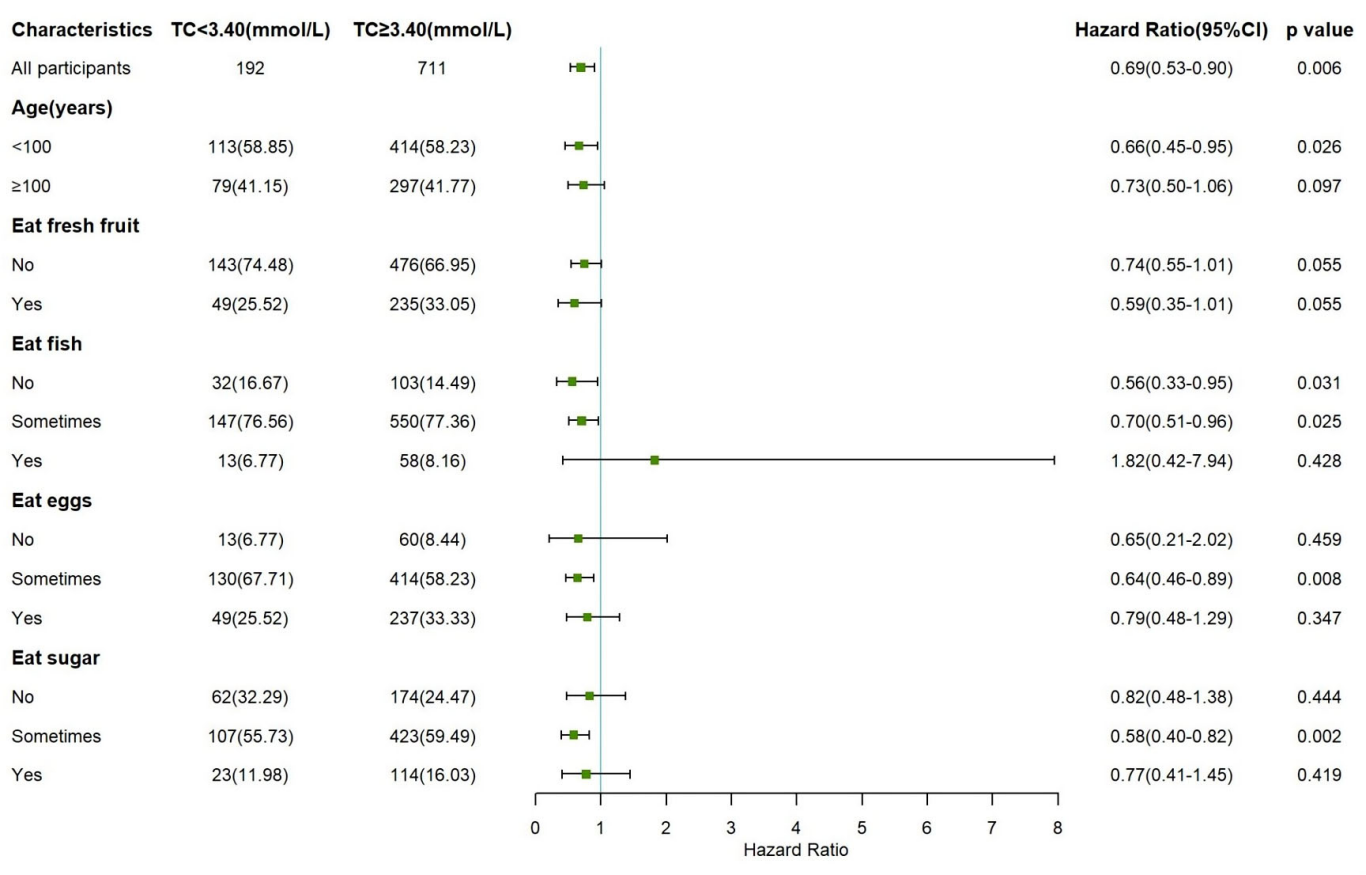
Forest plots showing subgroup analysis of the association between total cholesterol and all-cause mortality according to total cholesterol ≥3.40 mmol/L compared with <3.40 mmol/L (Reference group).

Subgroup analysis was performed according to age, consumption of fresh fruit, fish, eggs, sugar (all of which were all-cause mortality potential influencing factors with significant associations in the multivariate analysis). In the subgroup analysis, participants with TC levels of ≥3.40 mmol/L had a significantly lower risk of all-cause mortality than participants with TC levels of <3.40 mmol/L only in the subgroup with age<100 years, sometimes consumption of fish, eggs, sugar. The results of the subgroup analysis are shown in the forest plots in **Figure 3**.

### Survival analysis

**Figure 4:**
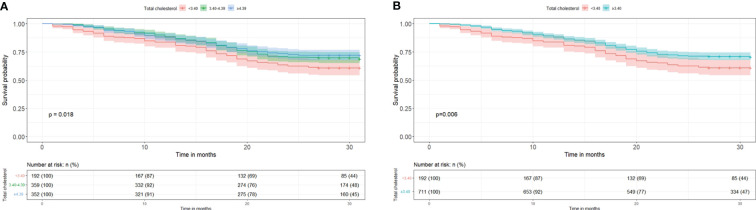
Kaplan–Meier survival curve estimates for all-cause mortality according to total cholesterol category. **(A)** Total cholesterol was divided into <3.40, 3.40-4.39, and ≥4.39 mmol/L. **(B)** Total cholesterol was divided into <3.40 and ≥3.40 mmol/L.

The Kaplan–Meier survival curves for all-cause mortality according to TC category are shown in **Figure 4**. Compared with participants with TC < 3.40 mmol/L, participants with TC 3.40 – 4.39 mmol/L and ≥ 4.39 mmol/L had a significantly higher survival probability (*P* = 0.018). In addition, compared with participants with TC < 3.40 mmol/L, those with TC ≥ 3.40 mmol/L had a significantly higher survival probability (*P* = 0.006).

## Discussion

The present findings showed that a lower TC level was associated with elevated all-cause mortality risk in a population of oldest-old adults (aged ≥85 years). When TC level was used as a continuous variable, the mortality risk increased by 12% with each 1 mmol/L reduction in TC. This is consistent with findings from several previous studies, which demonstrated that a low TC level was a risk factor for all-cause mortality in older people (9, 10). This could be explained by the increased risk of non-cardiovascular mortality (e.g., from cancers and infections) (16). After identifying this inverse association, we further explored the lower limit of TC and found a continuous increment of all-cause mortality risks when TC levels fell below 3.40 mmol/L. This indicates that a TC level <3.40 mmol/L is associated with higher mortality risk among oldest-old adults. To our knowledge, this is the first study to clarify the lower TC cutoff point in this population using the restricted cubic splines method. TC levels decline with age, and it has been suggested that lower TC levels are associated with frailty and chronic disease in seniors (17), which further increases mortality risk.

Some researchers suggest that higher TC levels are associated with better nutritional and chronic health status in the oldest old population; thus, individuals with elevated TC levels are more likely to live longer (18). In the present study, we attempted to exclude the potential effect of dietary or physiological factors by including these variables in the model. In our study, self-reported diabetes, heart disease and stroke were not significantly associated with all-cause mortality, and thus not included as covariates in the final multivariable models. The predictive value of traditional risk factors for mortality may diminish in the oldest old compared to younger populations, as supported by some previous studies (19, 20). As for dietary factors, fresh fruit consumption and fish consumption were protective factors in the multivariable model, whereas daily consumption of eggs and sugar were risk factors for all-cause mortality, which is consistent with previous findings (21–24). Adjustment for dietary behaviors and chronic health conditions did not alter the protective effect of TC on all-cause mortality, indicating that the association between TC and all-cause mortality is independent of nutritional status. Although the biological pathways that link TC to mortality are poorly understood, several mechanisms may explain this inverse association. For example, blood lipids, which are an important component of cell membranes, may affect cell electrophysiology by modulating the distribution and function of some ion channels (25). Low TC levels may contribute to the pathogenesis of some common diseases in older people, such as atrial fibrillation (26). Another potential mechanism is that TC may regulate inflammatory markers such as C-reactive protein and attenuate the biological response to inflammation (27). Therefore, individuals with low TC levels may be more vulnerable to physiological disorders because of enhanced inflammation (28).

The Kaplan–Meier curves showed that during the 3-year follow-up period, individuals with TC levels of 3.40–4.39 mmol/L and ≥4.39 mmol/L had 10.1% and 13.0% higher 2-year survival probabilities, and 6.8% and 12.3% higher 3-year survival probabilities than those with TC levels of <3.40 mmol/L. When we further explored the discrepancy between the 3.40–4.39 mmol/L and ≥4.39 mmol/L groups, in both of which TC served as a protective factor, we found no significant between-group difference in all-cause mortality risk. Previous research suggests that individuals of advanced age may be a select group that has lower TC, because individuals who are susceptible to physiological abnormalities induced by high TC die earlier (10). This is consistent with the distribution of TC levels in the present study, which shifted toward the left. Higher TC levels are associated with increased cardiovascular risk, and the <200 mg/dL (5.18 mmol/L) criteria has been proposed by the National Cholesterol Education Program guideline as a reference for older people to improve survival (7). However, our findings contribute to the growing body of evidence challenging the “lower is better” paradigm for cholesterol levels in older adults, and provide the possibility to limit the optimal TC level between 3.40–5.18 mmol/L. In sum, our study provides valuable data to inform the development of such age-specific guidelines and highlights the need for further research to validate these findings.

There are several study limitations that should be acknowledged. First, the sample size was relatively small and the follow-up period was short owing to the difficulty of obtaining blood samples from the oldest old population. Although our study is one of the largest to date focusing on this population in China, additional studies are needed with larger sample sizes and longer follow-up periods to further investigate the relationship between TC and all-cause mortality in oldest-old adults. Second, comorbidities were self-reported, which might lead to recall bias and underestimation, especially in the oldest old. It thus potentially attenuated the associations between self-reported comorbidities and mortality. Third, although dietary behaviors and chronic health status were included in the multivariable model, other biochemical indexes such as inflammatory markers may also affect the association between TC level and all-cause mortality. Future research should take into account a wider range of potential confounding factors. Fourth, although the data were drawn from a national longitudinal study of older people, they are not representative of the general population and younger seniors. However, the results may still have important implications for the ongoing search for longevity. Besides, we did not obtain information about death-specific mortality, and so could not determine whether lower TC levels are associated with increased mortality risk of cancers, infections, and other diseases. Finally, the last limitation is the lack of interaction tests for the subgroup analyses due to insufficient statistical power with the current sample size. Larger studies are warranted to formally assess potential effect modifiers. Alternatively, future studies could consider applying Bayesian methods to obtain more stable estimates of interaction effects in the presence of low event rates within subgroups.

## Data availability statement

The original contributions presented in the study are included in the article/supplementary material. Further inquiries can be directed to the corresponding authors.

## Ethics statement

The studies involving humans were approved by research ethics committee of Peking University (IRB00001052-13074). The studies were conducted in accordance with the local legislation and institutional requirements. The participants provided their written informed consent to participate in this study.

## Author contributions

FH: Data curation, Formal Analysis, Methodology, Visualization, Writing – original draft. ZW: Data curation, Formal Analysis, Methodology, Visualization, Writing – original draft. YL: Data curation, Methodology, Writing – original draft. YG: Conceptualization, Formal Analysis, Methodology, Writing – original draft. SL: Conceptualization, Investigation, Methodology, Writing – original draft. CX: Conceptualization, Data curation, Investigation, Writing – original draft. YW: Data curation, Methodology, Resources, Writing – original draft. YC: Data curation, Methodology, Resources, Writing – original draft.
